# Flipper: An advanced framework for identifying differential RNA binding behavior with eCLIP data

**DOI:** 10.64898/2026.03.13.711628

**Published:** 2026-03-31

**Authors:** Keegan Flanagan, Shuhao Xu, Gene W. Yeo

**Affiliations:** 1 Bioinformatics and Systems Biology Program, University of California San Diego, 9500 Gilman Drive, La Jolla, California 92093, USA; 2 Department of Cellular and Molecular Medicine, University of California San Diego, 9500 Gilman Drive, La Jolla, California 92093, USA

## Abstract

**Motivation:**

Crosslinking and immunoprecipitation (CLIP) methods remain the gold standard for characterizing RNA binding protein (RBP) behavior. As a result, many researchers rely on CLIP to assess how treatments targeting RBPs alter binding patterns and regulatory activity. However, current tools for differential RBP binding analysis lack core features required for rigorous statistical inference, including proper normalization and appropriate handling of replicate experiments. Furthermore, existing approaches cannot adequately separate expression driven effects from true changes in RBP binding, complicating interpretation of differential analyses. Addressing these limitations is essential for producing reproducible and informative analyses of differential RBP binding.

**Results:**

Here we present Flipper, an application purpose built for the analysis of differential RBP binding. Flipper introduces several innovations that adapt the DESeq2 framework for robust differential analysis of eCLIP count data. These include integration of input controls to account for expression driven binding shifts, hierarchical normalization strategies that adjust for technical variation without confounding signal to noise ratios, and improved post-differential analysis tools. We demonstrate that Flipper exhibits high specificity when applied to real differential eCLIP data while also providing deeper biological insights. In addition, analyses of both real and simulated data indicate that Flipper achieves superior sensitivity and precision compared with existing approaches. Together, these results highlight Flipper as a robust and generalizable framework for differential RBP binding analysis.

## 1 Introduction

RNA-binding proteins (RBPs) modulate a wide range of cellular processes (Gerstberger et al., 2014;Hentze et al., 2018), including translation (Svitkin et al., 1996), RNA degradation (Hasan et al., 2014), and splicing (Lovci et al., 2013). As a result, RBPs play a crucial role in cellular regulation, and mutations or other defects in these proteins have been implicated in numerous genetic diseases and cancers (Lukong et al., 2008; Kechavarzi and Janga, 2014; Bardoni et al., 2001;Verkerk et al., 1991). Consequently, studies seeking to perturb RBP function through mutations or drug treatments have become increasingly common (Li and Kang, 2023;Bertoldo et al., 2023). This has created an urgent need for experimental and computational frameworks for studying changes in RBP binding across conditions.

Investigations of RBP–RNA interactions have long relied on cross-linking and immunoprecipitation (CLIP) methods. Although there are several variations of CLIP (Hafner et al., 2021),they share a common experimental framework. In these assays, RBPs are cross-linked to their RNA targets, and antibodies are used to immunoprecipitate the RBP of interest along with its associated RNA. The recovered RNA is then sequenced, and genomic regions with enriched read coverage are identified as putative binding sites using peak calling algorithms such as Piranha (Uren et al., 2012), DEWSeq (Schwarzl et al., 2024), and Skipper (Boyle et al., 2023).

Several methods and tools exist for performing differential analysis using CLIP data. Early approaches relied on performing peak calling in two conditions and designating peaks unique to each condition as differential. While simple and straightforward, the qualitative nature of this analysis has major shortcomings. This approach is hereafter referred to as the ad-hoc method. Quantitative alternatives include repurposing peak calling software for differential analysis (Uren et al., 2012), as well as published differential CLIP tools such as dCLIP (Wang et al., 2014) and DeepRNA-reg (Sekhon et al., 2025). Although these methods represent conceptual improvements over the ad-hoc approach, they also have substantial limitations. For example, dCLIP and DeepRNA-reg are unable to accommodate multiple CLIP replicates, while repurposed peak callers are applied to inference tasks beyond their original design scope. Furthermore, all of these methods lack a key feature required for rigorous differential binding analysis—control for RNA expression.

The number of RNA fragments recovered by CLIP at a given genomic region reflects both the strength of RBP–RNA binding and the abundance of the RNA substrate. Without a strategy to disentangle these effects, changes in pulldown cannot be unambiguously attributed to altered binding versus altered expression. Because many perturbations that influence RBP binding also impact RNA abundance, this confounding of expression and binding poses a fundamental obstacle to differential binding analysis.

Most CLIP protocols do not provide direct information on RNA expression, making it difficult to control for RNA substrate abundance during differential analyses without additional RNA-seq data. Even when such data are available, careful consideration is required to ensure that RNA-seq and CLIP measurements are comparable. Enhanced CLIP (eCLIP) (Van Nostrand et al., 2016) offers a potential solution to this challenge. eCLIP includes the collection of a size-matched input (IN) fraction alongside the immunoprecipitate (IP) fraction. Although this IN data is traditionally used for background correction, it can also be leveraged to control for RNA expression in differential CLIP analyses.

In this study, we present Flipper, a Snakemake-based pipeline designed to leverage the unique structure of eCLIP data for differential RBP binding analysis. Flipper integrates IP and IN count data within a unified framework, enabling explicit modeling of changes in RBP binding while accounting for underlying RNA expression. Specifically, Flipper extends the DESeq2 (Love et al., 2014) methodology to test interactions between IP and IN counts across conditions, allowing differential binding to be interpreted as changes in pulldown normalized to transcript abundance rather than changes in IP signal alone. DESeq2 has been previously applied to the analysis of eCLIP data (Schwarzl et al., 2024), and to differential analysis with similar assays such as methylated RNA immunoprecipitation sequencing (MeRIP-seq) (Tang et al., 2021). However, DESeq2 has yet to be used for differential eCLIP analysis.

To properly apply DESeq2 to differential eCLIP analysis, Flipper introduces several innovations. These include using gene-wise aggregated IN values for increased statistical power, and implementing a hierarchical normalization strategy that independently accounts for pulldown efficiency and sequencing depth in the IP fraction. For computational efficiency and robustness, Flipper operates downstream of the Skipper peak caller (Boyle et al., 2023), restricting analysis to genomic windows exhibiting significant IP enrichment in at least one condition. Together, Skipper and Flipper form a complete end-to-end pipeline for differential eCLIP analysis. Using both real and simulated datasets, we demonstrate that Flipper recapitulates established differential RBP binding results while consistently outperforming existing approaches.

## 2 Materials and methods

### 2.1 Data preprocessing and window-level quantification

Flipper operates directly on the outputs of the Skipper peak caller. Briefly, Skipper partitions the transcriptome into approximately 100 base pair windows while ensuring that windows do not cross feature boundaries (e.g., intron exon junctions). For each window, Skipper quantifies IP and IN reads and applies a beta-binomial framework to identify windows in which IP read counts are significantly enriched over IN. For additional details on Skipper, see the original publication (Boyle et al., 2023).

**Figure 1:**
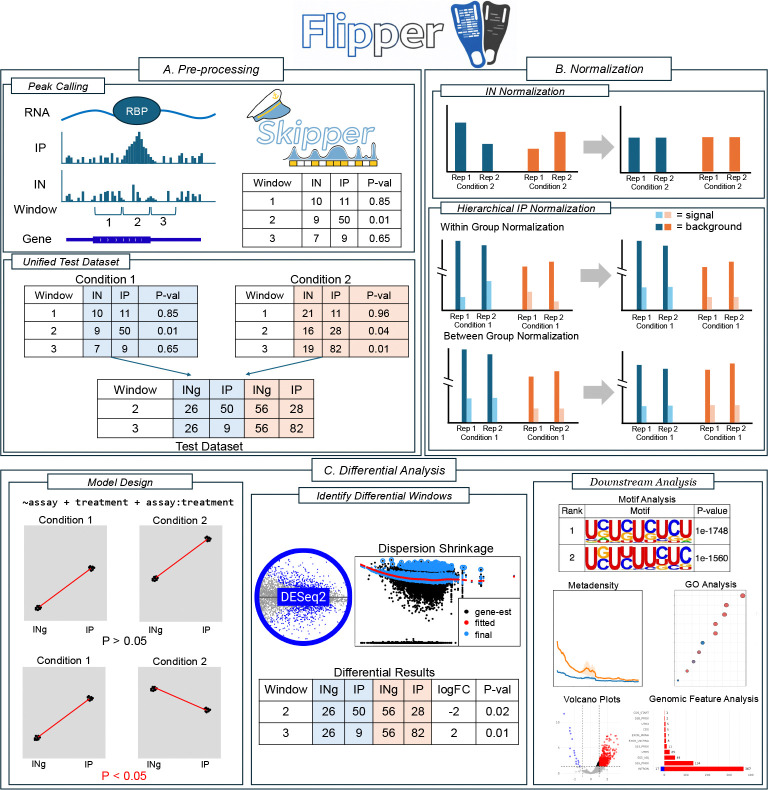
Overview of Flipper. The workflow consists of three major stages. A) Preprocessing and peak consolidation. Candidate binding sites are identified in each condition using Skipper and merged into a unified count matrix containing window-level IP counts and gene-level input (INg) counts. B) Independent normalization of INg and IP signals. INg counts are normalized using the median-of-ratios method, whereas IP counts undergo hierarchical normalization to separately account for enriched and background regions. C) Differential analysis using a DESeq2 framework that models assay type and condition to detect changes in IP signal relative to INg between treatments. Significant sites are subsequently summarized through downstream analyses.

Flipper then constructs a unified dataset containing IP and IN read counts for all windows that exhibit significant IP enrichment in at least one treatment group (Fig. 1A). We first attempted to use window-level IN values to control for expression similarly to what has been done for MeRIP-seq differential analysis (Tang et al., 2021; Guo et al., 2022). However, MeRIP-seq and eCLIP differ in how IN background is collected. MeRIP-seq uses total RNA as the IN (Meyer et al., 2012) while eCLIP uses size-matched IN that has undergone the same size selection as the IP (Van Nostrand et al., 2016). As a result, eCLIP IN is sparser than MeRIP-seq IN, making window-level IN counts too small to reliably estimate expression change. Flipper therefore aggregates IN read counts across all windows within a gene to obtain a gene-level input value (INg), which is used for expression control in downstream analyses.

### 2.2 Normalization framework

The general assumption of normalization methods applied to traditional differential RNA-seq analysis is that the vast majority of genes will remain unaffected, allowing identification of a discrete subset of treatment affected genes. (Love et al., 2014). When performing differential CLIP analysis, this assumption is often violated. A mutation in an RBP binding region or the attachment of a small molecule can lead to near global changes in binding affinity and pulldown efficiency. One possible solution is to use non-binding background regions to control for sequencing depth (Stark et al., 2011); however, this strategy does not account for differences in signal to noise ratios between IP replicates (Shao et al., 2012). Technical variation can affect both background and true binding signal, which can cause discordance in the signal to noise ratios across samples and replicates.

For example, variation in washing efficiency may increase background read counts in one IP replicate relative to another, whereas variation in pulldown efficiency may reduce reads at true binding sites in that same replicate. If normalization is based only on background reads, these opposing effects can distort the signal: true binding differences become exaggerated, variability increases, and sensitivity decreases (Fig. S1). To address this, we developed a hierarchical normalization strategy that performs the normalization in multiple stages, each addressing a distinct source of technical variation (Fig. 1B). IP data are divided into two groups: a large group containing background regions and a smaller group containing binding regions as identified by Skipper. In the first normalization step, scaling factors or offsets are estimated using only binding regions within individual treatment groups, using either median of ratios normalization (MOR) (Love et al., 2014) or EDAseq (Risso, 2011). This step assumes that overall binding levels between replicates within a treatment group are comparable, an assumption that is generally reasonable. In the second normalization step, scaling factors are estimated from background regions across all treatment groups and replicates using MOR. These background derived scaling factors are then averaged within each treatment group and combined with the binding derived scaling factors from the first step. This procedure aims to account for differences in sequencing depth between treatment groups without imposing assumptions about signal to noise ratios.

In contrast to IP libraries, IN libraries do not undergo immunoprecipitation and therefore do not exhibit binding-dependent signal to noise variation. Accordingly, all IN samples are normalized jointly using a conventional global scaling approach.

Although the EDAseq method offers additional normalization options, including adjustments for gene length and GC content, its increased complexity makes it difficult to guarantee that all available normalization options are appropriate for every dataset. As such, MOR is used as the default normalization method in Flipper. All results presented in this study were generated using hierarchical MOR normalization.

### 2.3 Differential binding analysis with DESeq2

The unified table containing both IP and gene-level input (INg) values for each window is used as input for DESeq2 using the following design formula:

∼assay+treatment+assay:treatment

where assay indicates whether counts originated from IP or INg, and treatment indicates whether counts are from the control or treatment group. This interaction design tests for changes in the relationship between IP and INg across conditions rather than testing changes in IP alone, thereby controlling for treatment associated changes in expression captured by INg.

Normalization is implemented using either the sizeFactors or normalizationFactors functions, depending on whether normalization was performed using MOR or EDAseq, respectively.

Windows with an adjusted p-value below 0.05 and an absolute log_2_ fold change greater than 1 were considered significantly differentially bound. The log_2_ fold change corresponds to the assay:treatment interaction term and represents the change in the IP/INg ratio between treatment groups.

### 2.4 Comparison with existing differential binding methods

To benchmark Flipper against existing approaches, we evaluated four alternative differential binding strategies: (i) an ad-hoc peak comparison method, (ii) a repurposed peak caller approach, and (iii–iv) two published tools, dCLIP and DeepRNA-reg. There exist many variants of both ad-hoc and repurposed peak caller approaches for differential binding analysis. Nearly any peak caller can be used in an ad-hoc framework, and any peak caller that incorporates IN background can, in principle, be repurposed for differential analysis. Here we chose Skipper as the peak calling method for both approaches to ensure consistent handling of the data. For the ad-hoc method, the same Skipper runs used as input for Flipper were analyzed directly. Windows identified exclusively in the control group were designated as sites of decreased binding, while windows identified exclusively in the treatment group were designated as sites of increased binding. For the repurposed peak caller approach, separate Skipper runs were performed in which the IP and IN inputs were replaced with IP data from the control and treatment groups. Because Skipper, like most peak callers, performs a one-directional test, two separate runs were required for each differential comparison. One run compared control IP to treatment IP to identify windows with increased binding, while a second run compared treatment IP to control IP to identify windows with decreased binding. This specific repurposing of the Skipper peak caller will be referred to as Diff-Skipper from this point onward.

Both dCLIP 1.0.0 and DeepRNA-reg 1.0 were downloaded from GitHub and run with default parameters. Both programs used the deduplicated BAM files generated by Skipper as input, with dCLIP requiring an additional step to convert the BAM files to SAM files. For both tools, experimental replicates were concatenated into a single alignment file per condition according to the recommendations of the dCLIP and DeepRNA-reg authors. DeepRNA-reg additionally requires a BED file specifying genomic regions to test. We initially attempted to run DeepRNA-reg on the entire transcriptome. However, DeepRNA-reg was initially built for analyzing binding to microRNA, and is prohibitively slow when applied to larger transcriptomic regions. As such, DeepRNA-reg was applied only to genomic windows found to be significant by Skipper.

Output formats and reported statistics differ substantially across methods. Comparisons were therefore limited to metrics available across all tools, such as the total number of differential regions identified. Estimates of differential binding strength or statistical certainty were not compared, as the metrics produced by each program are not directly analogous and are, in many cases, absent from one or more methods. For example, neither DeepRNA-reg nor dCLIP provides quantities analogous to adjusted p-values.

### 2.5 Differential RBP binding datasets

To date, there are no gold-standard differential eCLIP dataset in which all true differential binding events are known. As a result, evaluation of differential binding methods must rely on datasets with experimentally perturbed conditions for which biological effects have been independently characterized. Based on these considerations, three diverse eCLIP datasets were selected for analysis.

The first dataset was generated from an eCLIP experiment performed on the NONO RBP in 22Rv1 human prostate cancer cells (Kathman et al., 2023). In this experiment, cells were treated with the small molecule compound R-SKBG-1, which is reported to associate with NONO and stabilize RBP binding, as well as with a corresponding inactive compound (S-SKBG-1) and a DMSO control. Two replicates were collected for each of the three conditions. Raw FASTQ files are available through GEO under accession GSE198212.

The second dataset was generated from an eCLIP experiment performed on the DDX42 RBP in HCT116 cells (Lazear et al., 2023). In this experiment, cells were treated with the small molecule compound WX-02–23, which was designed to perturb splicing activity and DDX42 binding preferences, as well as with a corresponding inactive compound (WX-02–43) and a DMSO control. Two replicates were collected for each of the three conditions. Raw FASTQ files are available through GEO under accession GSE220185.

The third dataset was generated from an eCLIP experiment performed on the PUF60 RBP in MDAMB231 cells (Tankka et al., 2025). In this experiment, cells were made to express PUF60 with a cancer associated mutation at the L140P position of its RNA recognition motif 1 (RRM1) domain that was previously reported to decrease binding to PUF60’s canonical binding motif (Kralovicova et al., 2020), as well as with a corresponding wild type control. Two replicates were collected for each condition. Raw FASTQ files are available through GEO under accession GSE280899.

### 2.6 Simulation framework

Simulated read counts were generated for 30,000 genes across 8 replicates using negative binomial distributions following the approach described in (Love et al., 2014). For each gene, an intercept and treatment coefficient were sampled from normal distributions, and the baseline mean abundance was defined as 2^intercept^. gene-specific dispersions were computed as a function of this baseline mean and held constant across replicates. Replicates were divided evenly between control and treatment conditions, and the treatment coefficient *β* was incorporated through a log linear model to modulate the mean of the count distribution in treatment samples. Read counts for each replicate were then generated as independent draws from the corresponding negative binomial distribution. This approach preserved a shared underlying abundance for each gene while allowing replicate-to-replicate variability and condition specific effects. The resulting gene-level counts were subsequently down-sampled to more closely reflect the sparsity observed in eCLIP data. Simulation parameters, including the mean and standard deviation of the gene-level intercepts and treatment effects, were selected to closely replicate the distributions observed in the DDX42 eCLIP dataset (Fig. S2A).

Gene lengths for the simulation were drawn directly from the distribution of gene lengths seen in the hg38 genome. To ensure that gene lengths and gene counts exhibited correlation similar to that observed in real data, we coupled simulated gene counts and gene lengths. Specifically, gene counts and gene lengths were independently ranked, and lengths were then assigned to genes by matching these rank orders. Gaussian noise was added to the rank mapping to control the strength of the association (strength = 0*.*3), thereby inducing a strong but imperfect correlation between gene count and length.

Counts were then partitioned into high read-depth active regions and low read-depth inactive regions within each gene in order to approximate the difference in read depth between exonic and intronic regions. Counts were then further distributed into fixed width windows (100 bp) using window weights drawn from a gamma distribution. This gamma distribution included a region-specific (active and inactive) peakiness parameter that controlled how evenly counts were distributed across all windows. All parameters were selected to closely reflect the window-level count distributions observed in real data (Fig. S2B).

At this stage, four replicates were designated as IN samples and four replicates as IP samples. IN replicates were left unmodified. Within the IP samples, 6,000 genes were randomly selected from a set of sufficiently expressed genes and designated as bound genes. For each bound gene, a baseline binding strength, *κ*, was drawn from a shifted gamma distribution, ensuring strictly positive binding effects while allowing variability in binding magnitude across genes. To model differential binding, a multiplicative treatment effect parameter, *ζ*, was applied to a subset of bound genes. Half of the bound genes were randomly designated as differential, while the remaining half were assigned no treatment effect (fold change = 1). For differential genes, the binding strength in treatment IP samples was multiplied by *ζ*, producing a consistent fold change between control and treatment conditions; this treatment effect was applied only to IP samples belonging to the treatment condition. For each bound gene, the expected number of binding-associated reads in each IP replicate was computed as the product of the gene-level count and the corresponding binding multiplier. The resulting values were then rounded to integers to generate pulldown counts. These pulldown counts were then added to a single window within the corresponding gene, and the true binding strength and *ζ* values for that gene were recorded for the calculation of quality metrics. Parameters were selected to produce IP count distributions comparable to those observed among significant windows identified by Skipper in real datasets (Fig. S2C). Because the primary goal of this simulation is to evaluate differential binding methods rather than peak callers, the pulldown model was designed to generate binding signal representative of what can be identified by the Skipper peak caller, rather than attempting to replicate an unknown true biological pulldown distribution.

## 3 Results

### 3.1 Overview of Flipper and analysis strategy

Flipper is designed to address two central challenges in differential eCLIP analysis: normalization of IP signal across conditions and control for expression driven changes. The pipeline begins with peak calling and preprocessing, after which only regions showing significant IP enrichment in at least one condition are retained for analysis (Fig. 1A). This ensures that only relevant regions with adequate basal binding levels are analyzed by Flipper. At this stage, window-level IN counts are replaced with gene-level INg counts for later use in expression control. IP and IN data are normalized separately, with IP normalization performed in two steps to account for both enriched and background regions (Fig. 1B). Differential analysis is then performed using a DESeq2 framework that jointly models assay type and experimental condition (Fig. 1C). Sites are classified as differentially bound only when changes in IP signal cannot be explained by corresponding changes in expression. Significant sites are reported genome wide and subjected to downstream analysis, including multiple visualization strategies.

### 3.2 Benchmarking differential binding specificity on eCLIP data

Both NONO and DDX42 datasets include vehicle and inactive compound control groups, enabling direct assessment of specificity and reproducibility in differential eCLIP analysis. Minimal differential binding is expected between the vehicle (DMSO) and inactive compound control groups (S-SKBG-1 or WX-02–43), while substantial overlap in sites is expected when comparing each control to the active treatment condition (R-SKBG-1 or WX-02–23).

**Figure 2:**
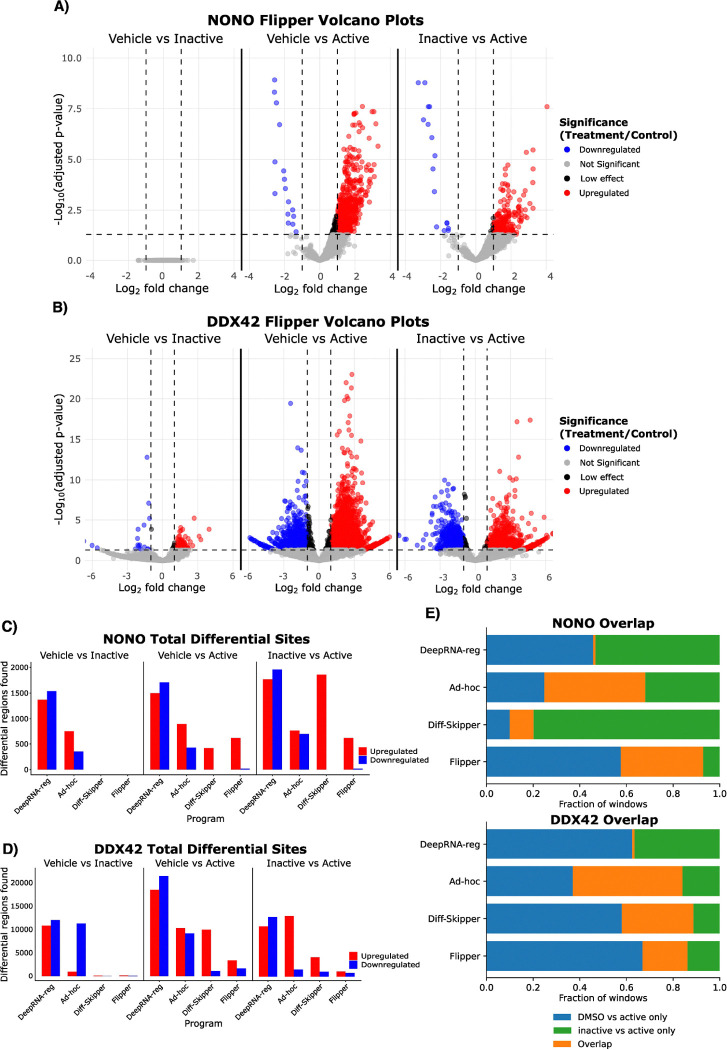
Method comparison for NONO and DDX42 eCLIP datasets. A-B) Volcano plots showing differential binding results for all comparisons in the NONO and DDX42 datasets, respectively. Minimal significant differences are observed between vehicle (DMSO) and the inactive compounds (S-SKBG-1 and WX-02–43), consistent with their expected lack of biological effect. C) Grouped bar plots summarizing the number of significantly up- and downregulated sites identified by each differential binding method. Upregulated sites correspond to increased binding following treatment, whereas downregulated sites indicate decreased binding. D) Overlap bar plots showing concordance of differential sites identified in the DMSO vs active compound and inactive compound vs active compound comparisons.

Differential analysis with Flipper detected no significant binding changes between the vehicle and inactive compound groups in the NONO dataset (Fig. 2A). In contrast, both vehicle vs active and inactive vs active comparisons exhibit a strong shift toward increased binding, consistent with prior evidence that R-SKBG-1 stabilizes NONO–RNA interactions (Kathman et al., 2023).

In the DDX42 dataset, a small number of sites are identified as differentially bound between vehicle and inactive compound conditions (Fig. 2B). However, these sites represent less than 1% of all tested regions, indicating a low false positive rate under null conditions (Supplementary table 1).

Across methods, Flipper and Diff-Skipper consistently identify few or no sites in vehicle vs inactive comparisons (Fig. 2C-D). In contrast, the ad-hoc method and DeepRNA-reg identify substantial numbers of differential sites under null conditions. Moreover, only Flipper and the Diff-Skipper recover the expected net increase in NONO binding following treatment, whereas the ad-hoc method finds a mix of increased and decreased binding and DeepRNA-reg identifies more sites with decreased binding than increased binding (Fig. 2C).

Consistency across contrasts was assessed by examining overlap between sites identified in vehicle vs active and inactive vs active comparisons. Flipper and Diff-Skipper show comparable levels of overlap, with Diff-Skipper performing slightly better in the DDX42 dataset and Flipper showing greater overlap in the NONO dataset (Supplementary table 1)(Fig. 2E). The ad-hoc method exhibits the highest overlap overall, but also identifies a large fraction of tested sites as significant, complicating interpretation of overlap as evidence of consistent site-specific detection (Supplementary table 1). DeepRNA-reg shows near-zero overlap between contrasts, highlighting the instability of its results (Supplementary table X)(Fig. 2E).

dCLIP identified hundreds of thousands to millions of regions as differentially bound even in vehicle vs inactive compound comparisons (Supplementary Table 1). Given the scale of these calls, their inclusion in Figure 2 would have obscured meaningful comparison with other methods. DeepRNA-reg similarly produced large numbers of differential calls under null contrasts and showed limited concordance between treatment comparisons. Owing to these discrepancies, subsequent quantitative benchmarking was restricted to Flipper, the ad-hoc method, and Diff-Skipper.

### 3.3 Comparison of differential binding sites across methods

We have found that Flipper and Diff-Skipper often produce similar trends when applied to vehicle vs active comparisons, as shown in Fig. 2. However, close inspection of the differential sites called by each method reveal clear differences in the count distributions underlying the calls.

**Figure 3:**
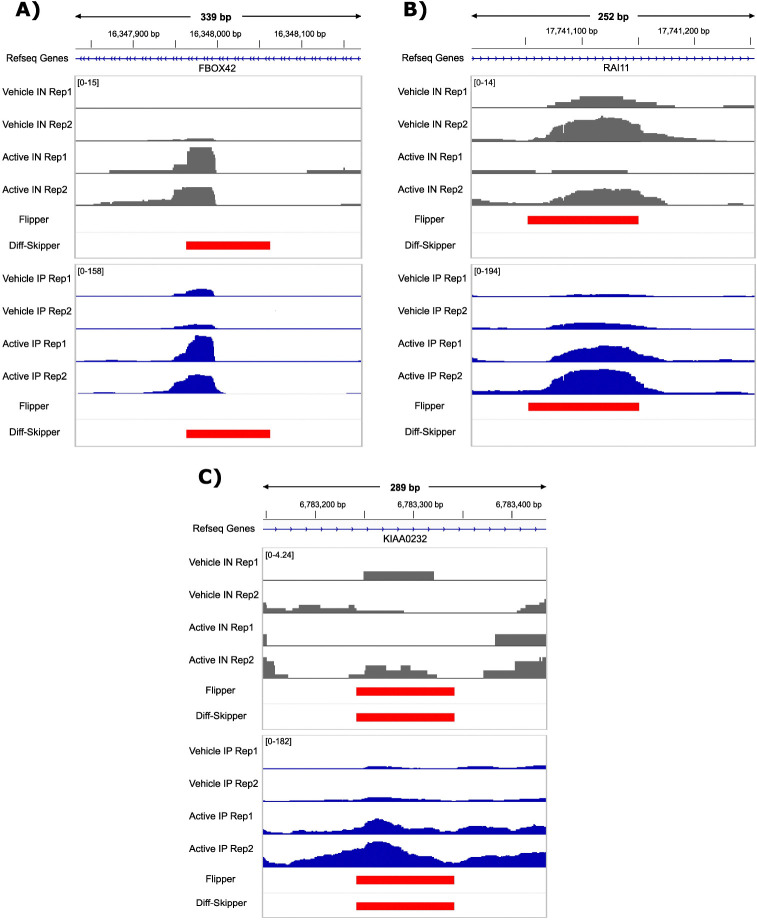
Genome browser tracks showing scaled read count distributions for example differential binding sites from the NONO dataset. Counts are scaled using size factors derived from Flipper’s hierarchical normalization scheme. Horizontal red bars indicate upregulated differential sites found by Flipper or Diff-Skipper. A) Site identified exclusively by Diff-Skipper, showing similar changes in IP and IN signal, consistent with expression driven effects rather than binding specific differences. B) Site identified exclusively by Flipper, showing an increase in IP signal around the binding site accompanied by a decrease in IN signal in the same region. C) Site identified by both Flipper and Diff-Skipper, showing an increase in IP signal with no apparent change in IN read density.

Because Diff-Skipper evaluates only IP read counts without incorporating IN signal, it often identifies differential peaks in regions where apparent changes in IP signal are closely mirrored by corresponding changes in IN. For example, the intronic region of FBOX42 shown in Fig. 3A shows increased read counts for the IP data, but a similar increase is seen for the IN counts. Thus, IN and IP read distributions in this region are consistent with an increase in RNA substrate rather than an increase in RBP binding.

In contrast, Flipper identifies sites like the example shown for the transcription factor RAI1 in Fig. 3B. In this case, IP read counts increase following treatment while IN counts decrease, suggesting an increase in binding despite an apparent decrease in expression. This highlights Flipper’s ability to identify changes in the relationship between IP and IN signal that suggest differential RBP binding activity.

Flipper and Diff-Skipper identify identical sites only when the change in IN between control and treatment groups is marginal, like the example shown in Fig. 3C.

These examples illustrate the fundamental distinction between Flipper and methods that rely solely on IP counts. IP only approaches may attribute expression driven changes to altered binding, while Flipper identifies sites where changes in the IP signal are not explained by a corresponding change in expression.

### 3.4 Flipper visualization enable downstream interpretation of differential binding

dCLIP, DeepRNA-reg, and the ad-hoc method produce relatively simple outputs, typically consisting of tables listing upregulated and downregulated binding sites, with dCLIP additionally providing BED files marking differential regions across the genome. Repurposed peak callers like Diff-Skipper often generate a larger number of visualizations; however, these outputs are primarily designed to support peak detection rather than differential analysis. In contrast, Flipper performs additional downstream analyses and produces intuitive visualizations designed to facilitate interpretation of differential binding results. The following examples are drawn from the PUF60 dataset comparing wild-type PUF60 to the L140P mutant, which has previously been shown to alter RNA binding activity (Tankka et al., 2025).

Flipper generates summary statistics for each gene containing at least one significantly differential binding site. For each gene, Flipper reports Fisher combined p-values and total (summed) log-fold change across all significant sites, which emphasize genes with large aggregate changes. Flipper additionally provides the minimum p-value per gene along with the corresponding log-fold change, highlighting genes driven by strongly differential individual sites. Gene-level summaries and associated visualizations are shown in Fig. S3.

**Figure 4:**
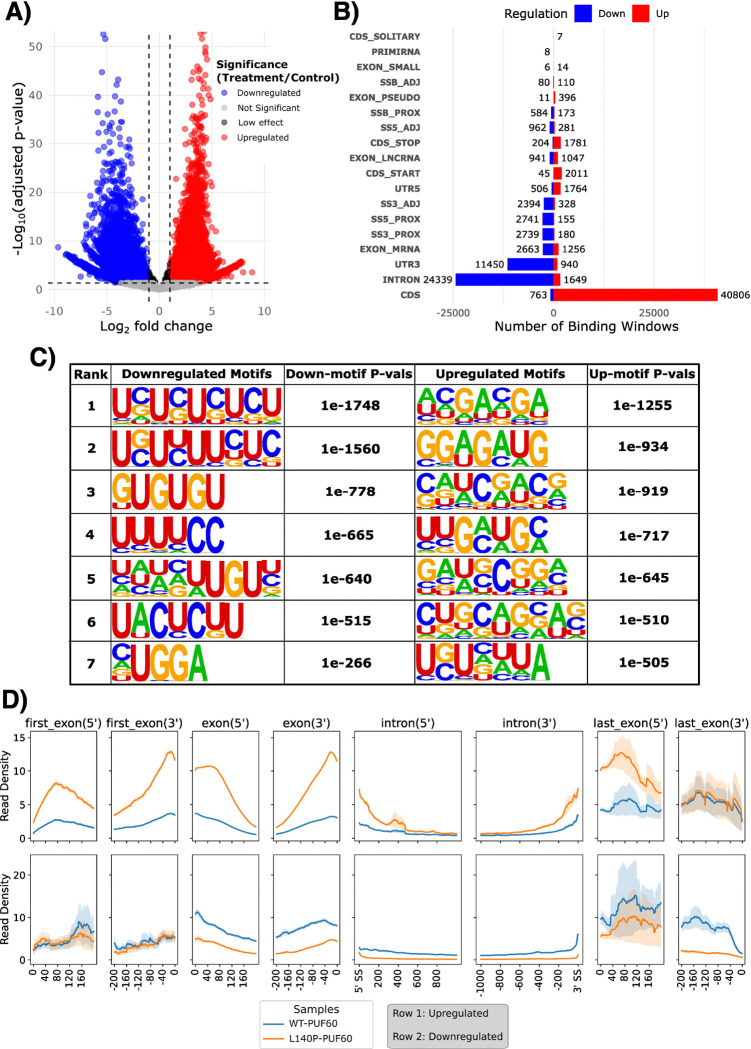
Flipper-generated visualizations for the PUF60 eCLIP dataset. A) Volcano plot showing widespread increases and decreases in binding following the L140P mutation. B) Diverging bar plot summarizing genomic feature enrichment, highlighting decreased binding within intronic and 3′ UTR regions and increased binding within coding sequences (CDS). C) HOMER motif analysis of up- and downregulated windows, with several UC-rich motifs enriched among downregulated sites. D) Metadensity plots showing relative read density across genomic regions containing significantly up- or downregulated binding sites in control and treatment conditions. Read density is estimated using the relative information (RI) metric from the original metadensity program. As expected, regions with upregulated binding sites exhibit increased treatment read density, whereas regions with downregulated binding sites show the opposite trend.

Flipper also generates a volcano plot summarizing the distribution of effect sizes (Fig. 4A) and a diverging bar plot showing the distribution of up and downregulated binding sites across genomic features (Fig. 4B). Consistent with prior reports, Flipper analysis finds the L140P mutation is associated with decreased binding at canonical intronic PUF60 sites. Notably, we also observe an increase in binding within coding sequence (CDS) regions. While *in vitro* studies suggest that the mutation reduces binding to canonical UC rich motifs (Kralovicova et al., 2020), its broader in vivo effects remain less well characterized. Motif analysis using HOMER (Heinz et al., 2010) further supports this distinction: downregulated sites are enriched for the known UC rich PUF60 binding motif, whereas upregulated sites lack this enrichment and do not exhibit a clear alternative motif (Fig. 4C).

To further characterize this redistribution, Flipper adapts the metadensity framework (Her et al., 2022) to visualize eCLIP signal across genomic features containing differential sites. These metadensity plots show that the L140P mutant exhibits increased read density near both 5′ and 3′ splice sites in regions containing upregulated binding sites, particularly within exonic regions (Fig. 4E, top row). A similar trend is observed in features that contain downregulated binding sites, although the change is comparatively more subtle (Fig. 4E, bottom row).

Together, these visualizations indicate that, in addition to reducing binding at canonical PUF60 sites, the L140P mutation is associated with a redistribution of binding toward exonic regions flanking splice sites. While this finding arose from an exogenous expression system and the mechanistic basis of this shift remains to be determined, it illustrates how Flipper enables detection of broader changes in binding architecture beyond canonical motif loss.

### 3.5 Benchmarking on simulated datasets

Although analysis of real datasets provides insight into the behavior of each method, quantitative performance metrics require access to known ground truth. To directly assess performance under controlled conditions, we evaluated Flipper, the ad-hoc method, and Diff-Skipper using simulated data.

Simulated eCLIP count data were generated under the assumption that IP pulldown levels depend on both the strength of the binding interaction and the expression level of the bound gene. Each simulated experiment consisted of eight replicates: four IN and four IP samples distributed across control and treatment groups. In the treatment condition, half of the binding sites were modulated by a treatment specific binding effect *ζ* corresponding to a 2, 3, or 4 fold change. Expression levels were either constant between control and treatment groups (*β* = 0) or allowed to vary under treatment (mean *β* = 0, SD *β* = 2). All other simulation parameters were selected to produce count distributions resembling those observed in the DDX42 eCLIP dataset (Fig. S2). Additional simulation details are provided in Section 2.6.

The performance of each differential binding method was evaluated using sensitivity and precision. Sensitivity was defined as the fraction of true differential binding sites correctly identified by the method, while precision was defined as the fraction of sites called significant that were truly differential. For each combination of treatment parameters, five replicate simulations were performed to assess the consistency of these performance metrics.

**Figure 5:**
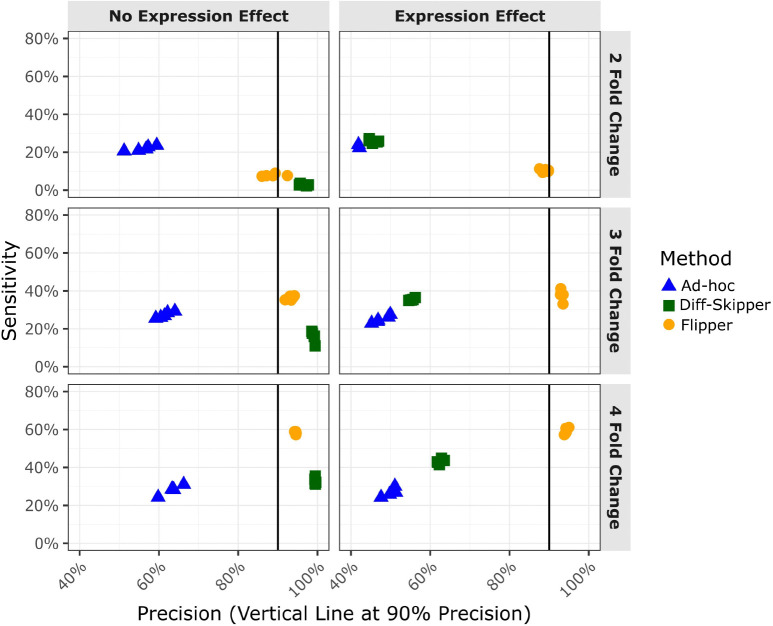
Sensitivity and precision of differential binding methods across simulated combinations of binding and expression treatment effects. Flipper performs comparably to or modestly better than alternative methods when expression effects are absent, and shows substantially improved performance when expression changes are present, reflecting its ability to distinguish binding-specific shifts from expression-driven differences.

Figure 5 compares the sensitivity and precision between methods for all six combinations of treatment effects. Flipper’s sensitivity increases with the magnitude of the binding effect, ranging from approximately 10% at a 2 fold change to nearly 60% at a 4 fold change. The precision remains consistently high at around 90%, with a modest increase at larger effect sizes. Although sensitivity at lower effect sizes is modest, this likely reflects the inherent sparsity and variability of eCLIP count data, where true differential binding events may be difficult to detect without inflating false positives. The consistently high precision suggests that this tradeoff favors specificity in noisy experimental settings. Notably, Flipper’s performance is largely unaffected by treatment induced expression changes, demonstrating effective adjustment for expression effects.

The ad-hoc method exhibits relatively stable performance across conditions, with sensitivity between 20–30% and precision between 50–60%, consistently yielding the lowest precision among the methods evaluated. In the absence of expression effects, the Diff-Skipper follows a trend similar to Flipper, showing higher precision but lower sensitivity. However, when expression changes are introduced, its precision declines sharply to approximately 60%, approaching that of the ad-hoc method.

## 4 Discussion

The analysis of differential RBP binding following treatment or mutation is becoming an increasingly important component of both drug development and disease research. Despite this growing demand, existing approaches remain limited in their ability to address the unique statistical and experimental challenges inherent to using CLIP data for differential analysis. Here, we introduce Flipper, a method designed to address key challenges in differential RBP binding analysis, including control for RNA expression and normalization strategies that account for signal-to-noise variation across samples. By leveraging the DESeq2 framework in conjunction with gene-level IN adjustments, Flipper consistently outperforms prior differential methods across both real and simulated datasets while also enabling expanded downstream analyses.

When evaluated on real datasets, Flipper exhibited low false positive rates in control to control comparisons and produced highly concordant results across control to treatment analyses. Furthermore, Flipper recapitulated previously reported biological findings, including increased binding in the NONO dataset and reduced binding to UC rich regions in the PUF60 dataset. Although repurposed peak callers such as Diff-Skipper produced comparable differential site counts, examination of uniquely identified sites indicated that many were driven by RNA expression changes rather than binding-specific effects.

Analysis of simulated data further underscored this limitation, with Flipper consistently outperforming alternative methods in the presence of treatment-induced changes in RNA expression, achieving superior sensitivity and precision. Although Flipper demonstrated reduced sensitivity for detecting weak changes in binding efficiency, this tradeoff reflects a deliberate emphasis on maintaining high precision in the context of sparse, high-variance eCLIP datasets.

Notably, the two published differential CLIP methods, dCLIP and DeepRNA-reg, did not perform as expected when evaluated on real eCLIP datasets. In the case of dCLIP, this behavior may reflect a violation of its core assumption that global binding levels remain stable across conditions. In many differential eCLIP experiments, treatments induce net increases or decreases in RBP binding, undermining this assumption and potentially biasing normalization. For DeepRNA-reg, one possible explanation is limited generalizability beyond its original training context. DeepRNA-reg was trained on a single HITS-CLIP dataset for the mouse microRNA mir-155 (Loeb et al., 2012) and subsequently evaluated on related microRNA CLIP datasets. Because microRNA binding events typically generate narrower peaks than those observed for most RBPs (Sekhon et al., 2025), models optimized for such signals may struggle to detect broader binding patterns characteristic of many eCLIP studies.

Several limitations of Flipper warrant consideration. As an extension of the DESeq2 framework, Flipper inherits constraints common to count based generalized linear modeling approaches, including read depth–dependent sensitivity that can lead to both overly sensitive and insufficiently sensitive results. In addition, the current implementation of Flipper only supports pairwise comparisons between two treatment groups and has not yet been extended to accommodate more complex experimental designs. Finally, the hierarchical normalization strategy used by Flipper assumes that, on average, pulldown efficiency does not differ systematically between treatment groups. Although this assumption is difficult to validate experimentally, it enables detection of global shifts in binding that would otherwise be obscured by conventional normalization approaches. More robust normalization may ultimately require incorporation of external spike-in standards, similar to those developed for ChIP-seq (Patel et al., 2024). The development of analogous spike-in strategies for eCLIP represents an important future direction for improving differential RBP binding analysis.

Despite these limitations, our results demonstrate that Flipper provides a robust and principled framework for differential RBP binding analysis. By integrating expression aware modeling with hierarchical normalization and detailed downstream analysis, Flipper enables rigorous identification and contextualization of condition specific changes in RBP binding. As differential eCLIP experiments continue to become more common, pipelines that explicitly disentangle binding from expression effects will be essential for accurate biological inference.

## Supplementary Material

Supplement 1
