# Supplementary material for: Flipper: An advanced framework for identifying differential RNA binding behavior with eCLIP data
Source: bioRxiv. 2026 Mar 31:2026.03.13.711628. Preprint. [Version 2] doi: 10.64898/2026.03.13.711628 (PMC13060198; doi:10.64898/2026.03.13.711628)
Supplement: Supplement 1 [file media-1.pdf]

Table 1: Summary of differential binding calls and overlap across methods, datasets, and contrasts.

Program	Dataset	Comparison	Total sites tested	Total significant sites	Overlapping sites	Jaccard index
Repurposed peak-caller	NONO	DMSO vs S-SKBG-1	Transcriptome	0	NA	NA
		DMSO vs R-SKBG1	Transcriptome	415	210	0.1020
		S-SKBG-1 vs R-SKBG-1	Transcriptome	1854		
	DDX42	DMSO vs WX-02-43	Transcriptome	94	NA	NA
		DMSO vs WX-02-23	Transcriptome	10982	3757	0.3035
		WX-02-43 vs WX-02-23	Transcriptome	5153		
Adhoc Method	NONO	DMSO vs S-SKBG-1	1928	1106	NA	NA
		DMSO vs R-SKBG1	2010	1324	852	0.4351
		S-SKBG-1 vs R-SKBG-1	2422	1486		
	DDX42	DMSO vs WX-02-43	15207	12347	NA	NA
		DMSO vs WX-02-23	27008	19614	11102	0.4735
		WX-02-43 vs WX-02-23	16585	14937		
DeepRNA-reg	NONO	DMSO vs S-SKBG-1	1517	2904*	NA	NA
		DMSO vs R-SKBG1	1667	3199*	68	0.0099
		S-SKBG-1 vs R-SKBG-1	1954	3718*		
	DDX42	DMSO vs WX-02-43	13777	22751*	NA	NA
		DMSO vs WX-02-23	23311	39815*	646	0.0103
		WX-02-43 vs WX-02-23	15761	23534*		
Flipper	NONO	DMSO vs S-SKBG-1	1517	0	NA	NA
		DMSO vs R-SKBG1	1667	631	240	0.3535
		S-SKBG-1 vs R-SKBG-1	1954	288		
	DDX42	DMSO vs WX-02-43	13777	130	NA	NA
		DMSO vs WX-02-23	23311	4962	1112	0.1931
		WX-02-43 vs WX-02-23	15761	1908		
dCLIP	NONO	DMSO vs S-SKBG-1	Transcriptome	1696513	NA	NA
		DMSO vs R-SKBG1	Transcriptome	1764717	1278698	0.5688
		S-SKBG-1 vs R-SKBG-1	Transcriptome	1516910		
	DDX42	DMSO vs WX-02-43	Transcriptome	774660	NA	NA
		DMSO vs WX-02-23	Transcriptome	795520	592200	0.6036
		WX-02-43 vs WX-02-23	Transcriptome	628789		

Table S1: * DeepRNA-reg is capable of finding multiple binding sites within a single tested window, allowing the total significant sites to be greater than the total tested sites.

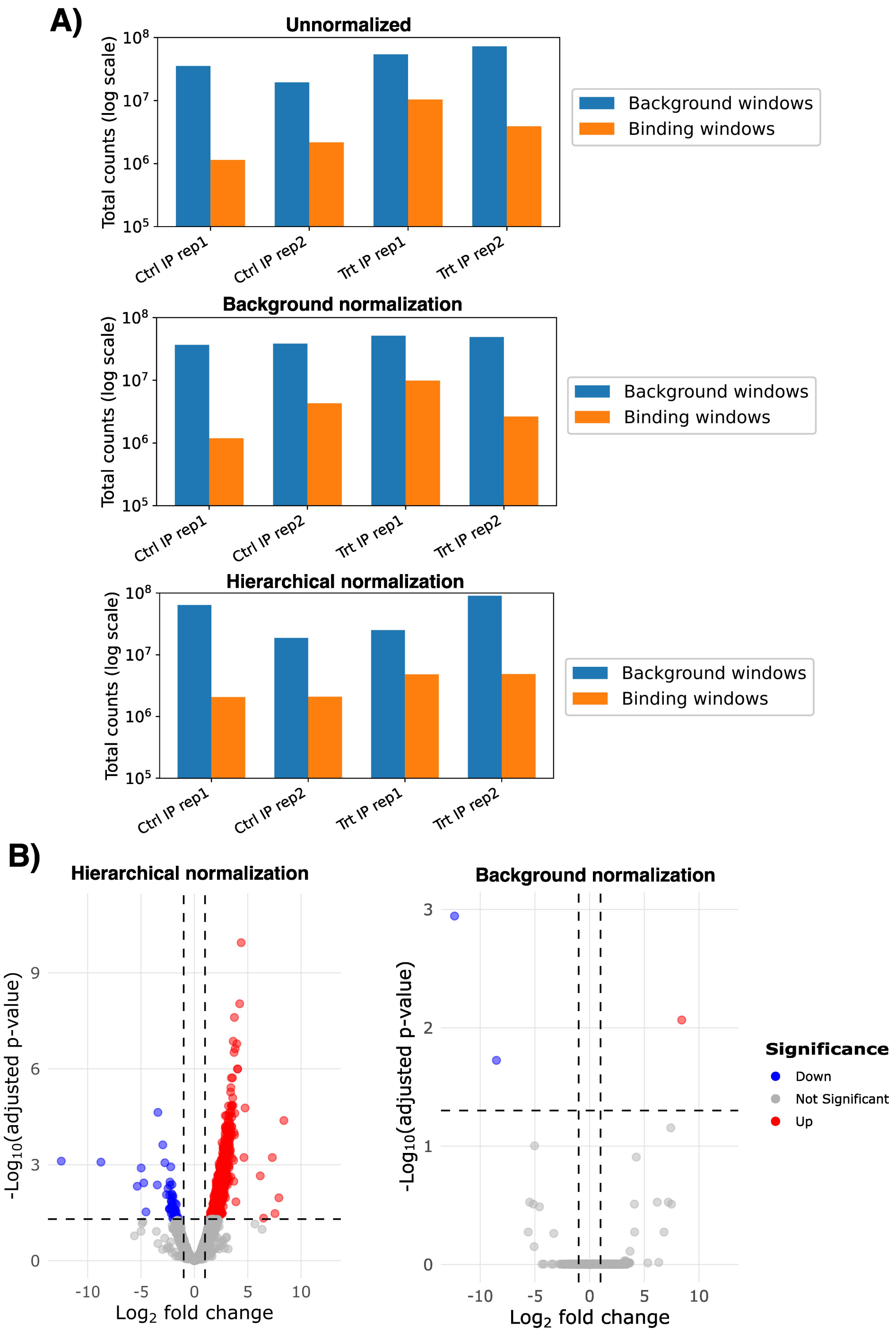

Figure S1: Demonstration of the importance of hierarchical normalization under signal-to-noise ratio shifts using simulated data. A) Comparison of replicate-level read counts across normalization strategies. Top: unnormalized total read counts, where relative differences between replicates are reversed when comparing signal and background windows. Middle: traditional background-based normalization, which amplifies replicate separation within binding windows. Bottom: hierarchical normalization, which restores comparable binding-window counts across replicates while preserving appropriate control of overall sequencing depth between treatment groups. B) Volcano plots comparing differential binding results obtained using hierarchical versus background normalization. Increased replicate variance introduced by background normalization markedly reduces the number of significant windows detected. Note that a net increase in binding is expected from this simulation.

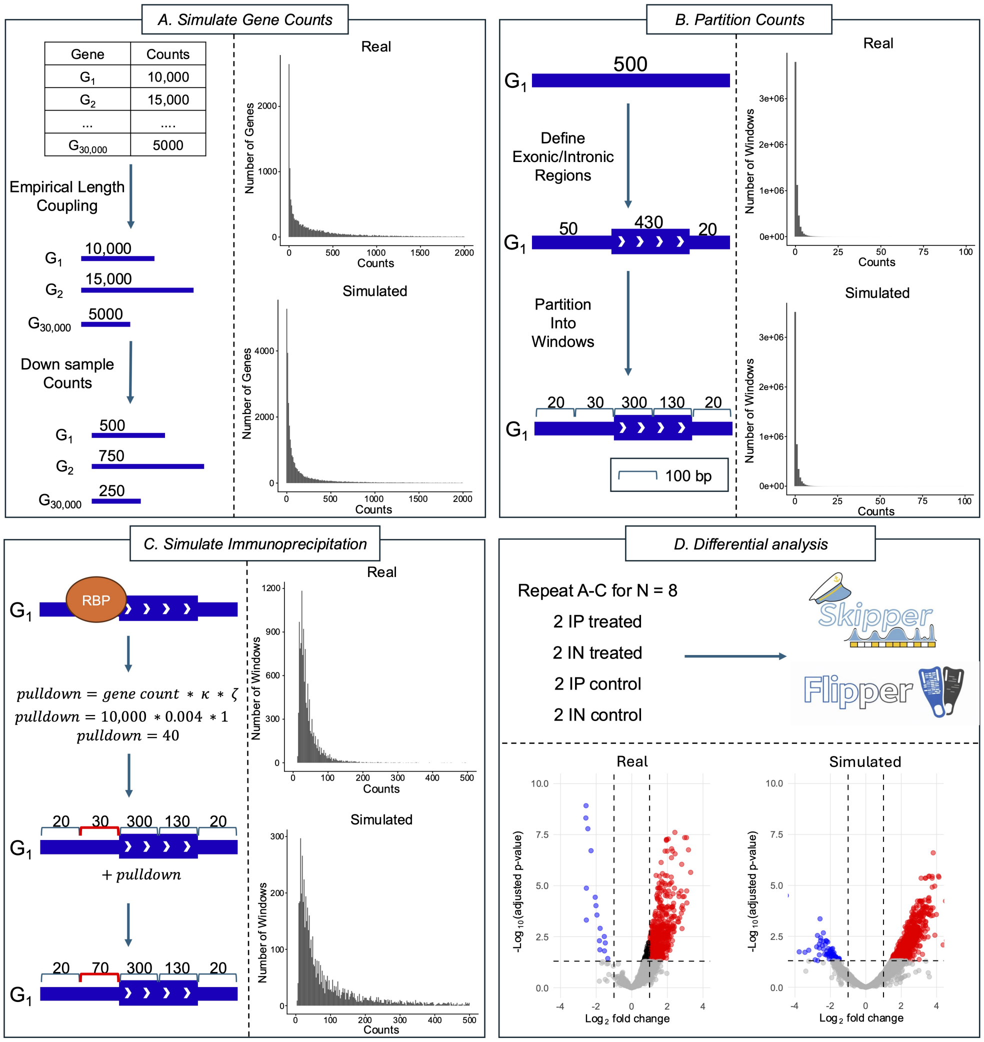

Figure S2: Overview of eCLIP simulation pipeline with comparisons between real DDX42 eCLIP data and simulated eCLIP data. Each panel shows a schematic of a major simulation step and a comparison between real and simulated data. A) Initial gene counts are generated from a negative binomial distribution and coupled to empirical gene lengths before being down-sampled to reflect the sparsity of eCLIP data. The distribution of simulated gene-level counts is similar to those observed in real data. B) Counts are divided into high and low read depth regions and then further partitioned into 100 bp windows. High similarity is observed between real and simulated window-level counts. C) Immunoprecipitation is simulated by adding a pulldown value to a specific window for each bound gene. The count distribution for simulated bound windows closely matches that seen in binding windows identified by Skipper in real data. D) The simulation is repeated to generate IP and IN data for both treatment groups, and these data are then subject to differential binding analysis. Volcano plots show similar distributions between real and simulated data.

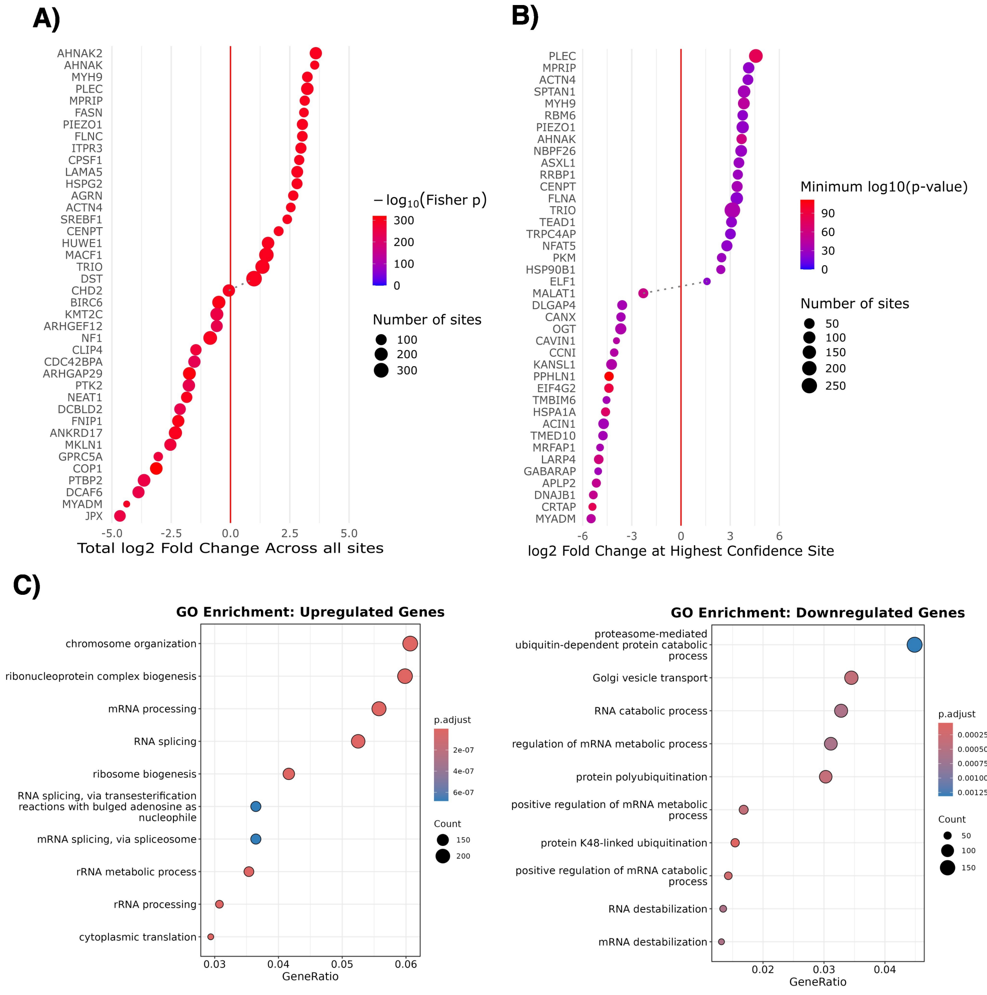

Figure S3: Gene-level analyses performed by Flipper for the PUF60 eCLIP dataset. A) Dot plot showing the top 40 genes ranked by combined Fisher's p-value across all significant sites per gene, with total $\log_2$ fold change summed across sites. B) Dot plot showing the top 40 genes ranked by the minimum p-value among significant sites per gene, with the corresponding $\log_2$ fold change at that site. C) Gene Ontology (GO) analysis of genes containing significant binding sites, stratified by net positive (upregulated) or net negative (downregulated) fold change in binding.
